# Differential Disruption of EWS-FLI1 Binding by DNA-Binding Agents

**DOI:** 10.1371/journal.pone.0069714

**Published:** 2013-07-22

**Authors:** Changmin Chen, Diane R. Wonsey, Madeleine E. Lemieux, Andrew L. Kung

**Affiliations:** 1 Department of Pediatric Oncology, Dana-Farber Cancer Institute and Boston Children’s Hospital, Harvard Medical School, Boston, Massachusetts, United States of America; 2 Department of Pediatrics, Columbia University Medical Center, New York, New York, United States of America; Johns Hopkins University, United States of America

## Abstract

Fusion of the *EWS* gene to *FLI1* produces a fusion oncoprotein that drives an aberrant gene expression program responsible for the development of Ewing sarcoma. We used a homogenous proximity assay to screen for compounds that disrupt the binding of EWS-FLI1 to its cognate DNA targets. A number of DNA-binding chemotherapeutic agents were found to non-specifically disrupt protein binding to DNA. In contrast, actinomycin D was found to preferentially disrupt EWS-FLI1 binding by comparison to p53 binding to their respective cognate DNA targets *in vitro*. In cell-based assays, low concentrations of actinomycin D preferentially blocked EWS-FLI1 binding to chromatin, and disrupted EWS-FLI1-mediated gene expression. Higher concentrations of actinomycin D globally repressed transcription. These results demonstrate that actinomycin D preferentially disrupts EWS-FLI1 binding to DNA at selected concentrations. Although the window between this preferential effect and global suppression is too narrow to exploit in a therapeutic manner, these results suggest that base-preferences may be exploited to find DNA-binding compounds that preferentially disrupt subclasses of transcription factors.

## Introduction

Ewing sarcoma predominantly affects adolescent and young adults, representing almost 3% of pediatric cancers [1], [2]. While the overall cure for patients with non-metastatic disease is approximately 70%, patients with metastatic disease have less than 20% 5-year event-free survival [3]. Accordingly, there is a significant unmet need for improved therapies to treat Ewing sarcoma.

A majority of Ewing sarcomas contain a translocation that fuses the *EWS* gene on chromosome 22 to the *FLI1* gene on chromosome 11. The chimeric EWS-FLI1 oncoprotein alters the regulation of wild type FLI1 transcriptional targets [4], [5]. In a minority of cases, the *EWS* gene is fused to other ETS-family transcriptional factors such as *ERG* and *ETV1* [6], [7]. Previous studies have shown that depletion of EWS-FLI1 results in cell cycle arrest and apoptosis in Ewing sarcoma cells [8], [9], [10], indicating that EWS-FLI1 may be an attractive therapeutic target [11], [12]. Unfortunately, conventional drug discovery approaches have not been as successful in targeting transcriptional factors by comparison to other target classes such as kinases and receptors.

One way to attenuate transcription factor activity is to block binding to the cognate DNA targets. For example, polyamide-based compounds have been shown to be able to bind DNA in a sequence-preferential manner, and to block the binding of transcription factors such as NF-κB [13] and hypoxia-inducible factor [14]. Prior focused and genome-wide analyses have shown that EWS-FLI and FLI1 bind to distinct regions of the genome, and have identified a consensus cognate binding motif for EWS-FLI1 [15], [16], [17]. Using this knowledge, we designed a high-throughput screening (HTS) assay to identify compounds that block the binding of recombinant EWS-FLI1 to a cognate oligonucleotide target. We used this HTS assay to screen libraries enriched for bioactive molecules, demonstrating that a variety of DNA-binding agents disrupt binding of EWS-FLI1 to DNA. Although no EWS-FLI1-specific inhibitors were found, some compounds, such as actinomycin D, did demonstrate preferential disruption of EWS-FLI1 binding.

## Materials and Methods

### Cell lines and Materials

All Ewing sarcoma cell lines were obtained from American Type Culture Collection (ATCC) and cultured in DMEM with 10% FCS or RPMI-1640 with 15% FCS (Life Technologies). Rearrangement of the *EWS* locus was verified in all Ewing sarcoma cell lines by split probe fluorescence *in situ* hybridization (FISH) at Genzyme. CellTiter 96 non-radioactive cell proliferation ***assay*** **(** ***MTT*** **)** was purchased from Promega. Absolutely RNA ***microprep kit*** was obtained from Stratagene. SuperScript III one-step RT-PCR system with platinum *Taq* high fidelity, BL21 (DE3) and DH5 alpha competent cells were purchased from Life Technology. X-tremeGene HP transfection reagent was obtained from Roche. BugBuster Protein Extraction Reagent was obtained from Novagen. AlphaScreen streptavidin conjugated donor beads and glutathione conjugated acceptor beads were obtained from PerkinElmer. All oligos were synthesized by ***Integrated DNA Technologies.*** Normal rabbit IgG was purchased from Cell Signaling Technology. ChIP-IT Express Chromatin Immunoprecipitation Kits were purchased from Active Motif. iScript One-Step RT-PCR Kit for Probes and iQ SYBR Green Supermix were obtained from Bio-Rad. Chemicals were purchased from Sigma-Aldrich and EMD Chemicals. Miniprep and Maxiprep DNA purification kits, PCR purification kits, DNA Gel extraction kits were obtained from Qiagen. All restriction enzymes, calf intestinal alkaline phosphatase (CIP) and T4 DNA ligase are purchased from New England Biolabs. p53-GST fusion protein expression plasmid was a kind gift from Dr. Guangchao Sui of Wake Forest University. 10X Tris Buffered Saline (TBS) was purchased from Boston BioProducts**.**

### AlphaScreen Assay

An AlphaScreen assay for binding of recombinant EWS-FLI1 to DNA was previously described [18]. Briefly, recombinant EWS-FLI1 and p53 were synthesized in bacteria transformed with pGex-6P-1-EWS-FLI1 or pGex-6P-1-p53, and bound to AlphaScreen acceptor beads. A biotinylated oligonucleotide containing two copies of the EWS-FLI1 binding motif or p53 binding motifs was bound to AlphaScreen donor beads. For EWS-FLI1, the oligo sequence was: ATGACACTGACCCGCCTACTACCGGAAGCGACCGGAAGCGCCCATCGCTC. The p53 binding oligo sequence was: GTCCAGTTAGTCTCCGATAACGCTGCCTAAGGTCACGAATTGACATAGCCAATGCGCTGT. This assay was used to screen 5,200 compounds, as previously described [18]. IC_50_ values were calculated with the GraphPad Prism software package.

### Luciferase Reporter Assays

The NR0B1 promoter was amplified from human genomic DNA using the 5′ primer- aaagctagcttcctcttatgctgagaattc and 3′ primer - gccaagcttggcgcccgtagcccagttctg. NR0B1 promoter amplicon was cloned into the NheI and HindIII sites in the pGL.3Basic vector. The 5′ primer-aaagctagctgcaagtgggagctaaataaag was used to amplify a truncated NR0B1 promoter in which the entire EWS-FLI1 binding region (GGAA repeats) was deleted to construct pGL.3b-NR0B1del. All constructs were confirmed by sequencing. All other plasmids were previously described [18].

Cells were grown in 6 well plates to approximately 50% confluence. A total 3.06 µg of DNA (0.9 µg of pGL.3b-NR0B1 or pGL.3b-NR0B1del, 0.06 µg of pGL.3b-UbC-RL and 2.1 µg of pCMV-tag4a-EWS-FLI1 or pCMV-tag4a empty plasmid) was mixed with 291 µl of serum free Opti-MEM medium. The mixtures were kept at room temperature for five minutes, then 9 µl of X-tremeGene HP was added. DNA and X-tremeGene HP mixtures were kept at room temperature for fifteen minutes, then 100 µl of the mixture was added to each well of a 6-well plate. Twenty four hours post-transfection, cells were lysed in 200 µl of lysis buffer and 10 µl of cell lysate was used for the dual luciferase assay.

Ewing sarcoma A673 cells at 50% confluence in 100 mm dishes were transfected with both 5.8 µg of pGL.3b-NR0B1 and 0.2 µg of pGL.3b-Ubc-RL reporter plasmids as above. Eight hours post-transfection, cells were trypsinized and 600 ul (2×10^5^) of cells were added to each well of 24 well plates. 5 µl of different concentration of compounds or DMSO control were added to each well. Each treatment was performed in triplicate. Cells were incubated for 16 hours, then cells were lysed in 100 µl of lysis buffer and 10 µl of cell lysate was used for the dual luciferase assay. Renilla luciferase activity was used as an internal control for normalization of nonspecific transcriptional inhibition or cytotoxic effects. Experiments were repeated three times. IC_50_ values were calculated using Prism (Graphpad).

### Chromatin Immunoprecipitation Assay

ChIP-qPCR was performed as previously described [18]. Briefly, cells were treated with compounds for 4 hrs, then crosslinked, lysed, and chromatin fragmented. After ChIP, quantitative PCR was used to quantify NR0B1, p53, and RPS26 loci, and fold enrichment was determined by the formula: Fold enrichment = 2χ(Input Ct-ChIP Ct)/2χ(Input Ct-IgG Ct).

### Quantitative RT-PCR

Duplicate wells of A673 cells were treated overnight with different concentration of actinomycin D. RNA was extracted with absolutely mRNA ***purification kit*** from Stratagene. 0.5 µg total RNA was mixed with 1 µl iScript Reverse Transcriptase, 10 µl of 2× RT-PCR reaction mix for probes from Bio-Rad, 1 µl of NR0B1, RPS26 and TP53 probes from Life Technologies and H_2_O up to 20 µl total volume. RT-PCR was performed in an iCycler iQ with 50°C for 10 minutes for one cycle; 95°C, 15 seconds, 60°C 40 seconds for 40 cycles. Each RNA sample was technically duplicated, and relative abundance was calculated by the delta Ct method.

### Cell Viability

Cells were seeded at a density of 1.5×10^5^/ml with 100 µl/well in 96 well plates and treated with actinomycin D at 158 nM, 50 nM, 15.8 nM, 5 nM, 1.58 nM, 0.5 nM and 0 (6 wells/treatment) for 44 hours. CellTiter 96 was used to assess cell viability after a 2 hr incubation at 37°C. IC_50_ values were calculated using Prism.

### Lentivirus Production and Viral Transduction

Lentiviral plasmids targeting FLI1- (clone ID TRCN0000005322, target sequence CGTCATGTTCTGGTTTGAGAT) and LacZ (clone ID TRCN0000072223, target sequence TGTTCGCATTATCCGAACCAT) were obtained from the RNAi Consortium and packaged according to the RNAi Consortium protocols. When A673 cells were 30 to 35% confluent in 100 mm dishes, growth media was removed and 5 ml fresh media added along with 5 ml of virus and Polybrene at a final concentration of 8 µg/ml. After 24 hours, media was replaced with 10 ml fresh growth media containing puromycin at 4 µg/ml. Cells were selected for 48 hours, then harvested for Western-blot and RNA extraction.

### Gene Expression Profiling

Ewing sarcoma A673 cells at 65% confluence were treated in duplicate with DMSO (0.2%) or 5 nM actinomycin D for 24 hours. RNA was extracted with an Absolutely RNA kit. 100 ng of total RNA was hybridized to Affymetrix Gene 1.0 ST Arrays (DFCI Microarray Core Facility). Array quality was verified before background correction, RMA normalization and log2 conversion using the Bioconductor R package *affy* (URL: http://www.bioconductor.org). Differential gene expression was determined using the *limma* Bioconductor package. Genes with a fold-change >2 and Benjamini-Hochberg-corrected [19] p-value <0.01 were retained for further consideration. Gene expression data have been deposited in GEO (GSE45414) and can be accessed at URL: http://www.ncbi.nlm.nih.gov/geo/query/acc.cgi?acc=GSE45414.

## Results

### Biochemical Disruption of EWS-FLI1 Binding

We developed a homogeneous AlphaScreen assay to assess the binding of recombinant EWS-FLI1 to DNA [18]. A parallel assay assessing binding of p53 to DNA was used as a counterscreen. Briefly, in these proximity assays, recombinant GST-EWS-FLI1 or GST-p53 was bound to glutathione-conjugated AlphaScreen acceptor beads, and a synthetic biotinylated oligonucleotide (oligo) containing tandem EWS-FLI1 or p53 binding sites was bound to streptavidin-conjugated AlphaScreen donor beads. Binding of GST-EWS-FLI1 or GST-p53 to the cognate oligo allows singlet oxygen transfer from donor to acceptor beads when excited at 680 nm, with resulting light emission between 520–620 nm.

**Figure 1:**
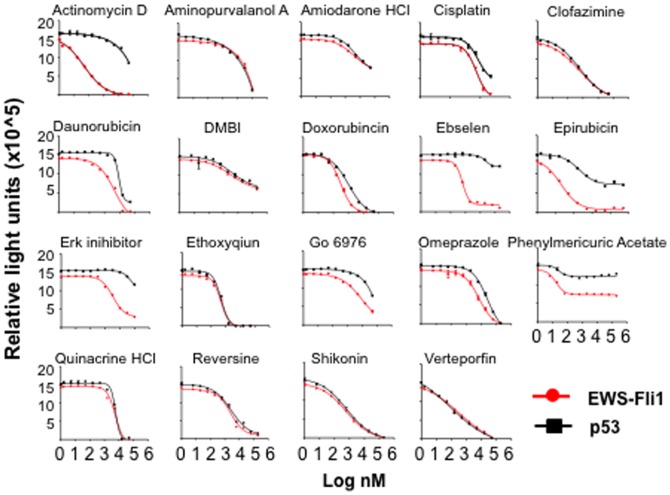
Biochemical disruption of EWS-FLI1 and p53 binding to DNA. Dose response for disruption of recombinant EWS-FLI1 (red lines) and p53 (black lines) binding to DNA in the presence of indicated compounds was measured using AlphaScreen proximity assays. Data plotted as mean +/− SD of triplicate samples and are representative of 2 independent experiments.

Using these assays, we screened 7 bioactive-enriched small molecule libraries, totaling 5,200 compounds (ICCB, Harvard Medical School), with an average Z’-factor of 0.84. A total of 19 compounds were found to disrupt the binding of EWS-FLI1 to its cognate DNA binding sequence with an IC50<10 µM (Figure 1, Table 1). Actinomycin D was found to have the greatest differential effect in the parallel assays, with an IC_50_ of 46 nM for EWS-FLI1 binding compared to >10,000 nM for p53 (Figure 1, Table 1). Actinomycin D, a chemotherapeutic agent isolated from soil bacteria [20], is a well known DNA-binding agent [21], [22], [23], [24], [25], [26], [27], [28]. Epirubicin, a DNA intercalating anthracycline and Ebselen, a mimic of glutathione peroxidase, also demonstrated some separation in disruption of EWS-FLI1 binding (IC_50_ 42 nM and 631 nM respectively) in comparison to p53 (IC_50_ 389 nM and >10,000 nM respectively). By contrast, several other DNA-binding chemotherapeutic agents were found to disrupt both EWS-FLI1 and p53 binding similarly (cisplatin, daunorubicin, doxorubicin). These results demonstrate that in a biochemical assay, there are differences between DNA binding agents in their ability to disrupt binding of EWS-FLI1 compared to p53.

**Table 1:** AlphaScreen Results.

	IC50 (nM)	
Compound	EWS-FLI1	p53
Actinomycin D	45	>10000
Aminopurvalanol A	1833	1202
Amiodarone HCl	5362	5288
Cisplatin	5391	8866
Clofazimine	202	203
DMBI	3371	3780
Daunorubicin	4482	8804
Doxorubincin	344	1216
Ebselen	631	>10000
Epirubicin	42	389
Erk inihibitor	3352	>10000
Ethoxyqiun	464	484
Go 6976	>10000	2813
Omeprazole	7328	26721
Phenylmericuric Acetate	16	25
Quinacrine HCl	7033	6055
Reversine	2416	2709
Shikonin	600	633
Verteporfin	342	86

### Inhibition of EWS-FLI1 Activity in Cells

To determine the effect of compounds on EWS-FLI1 activity in cells, we used a reporter assay comprised of the NR0B1 promoter, a prototypical EWS-FLI1-regulated gene [15], driving expression of firefly luciferase. Renilla luciferase driven by the Ubiquitin C promoter (UbC-RL) was used as an internal control. Confirming prior studies [15], the NR0B1 promoter was verified to be highly responsive to EWS-FLI1, whereas the UbC promoter activity was not subject to regulation by EWS-FLI1 (Figure S1).

**Figure 2:**
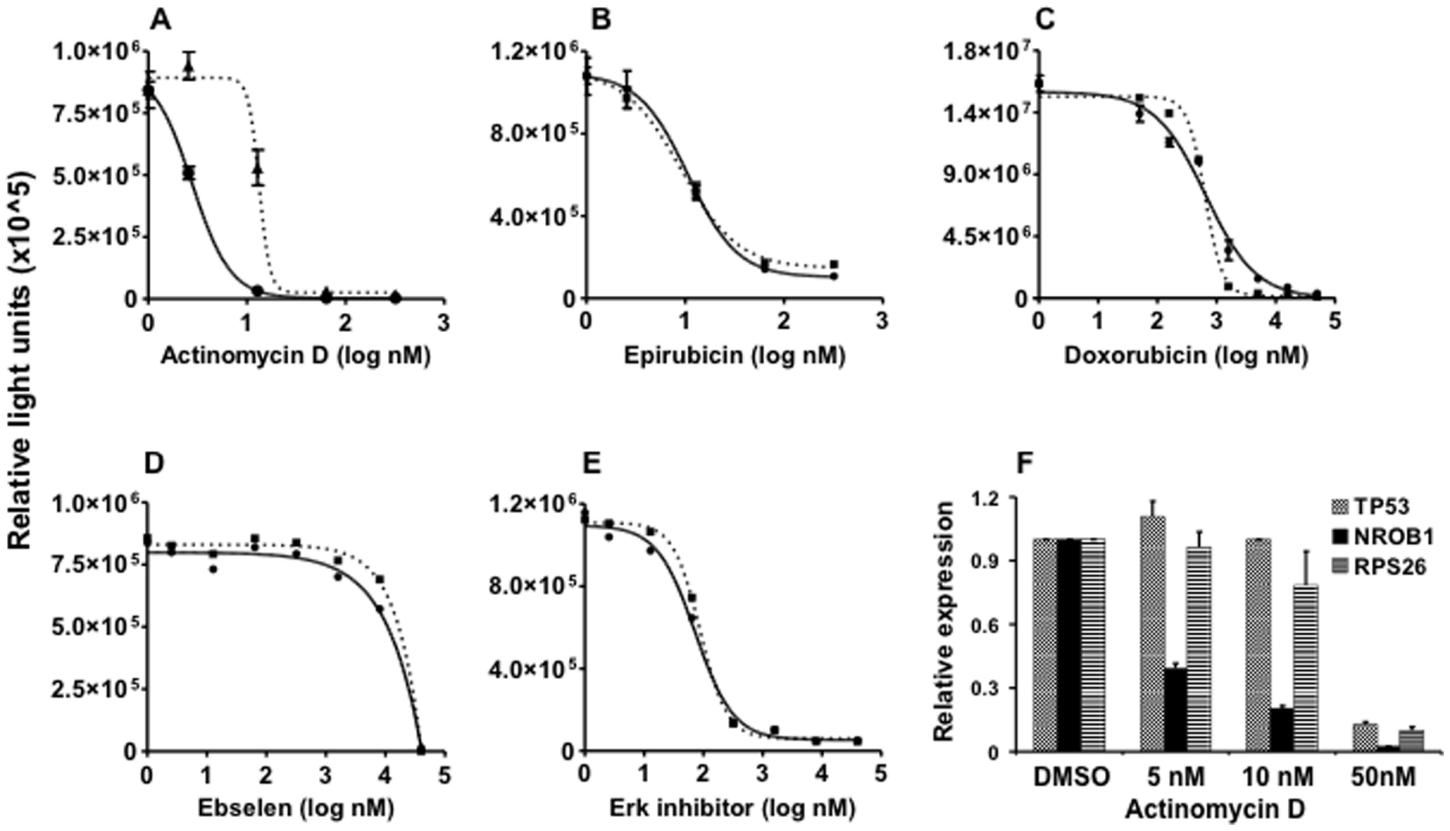
Effect of actinomycin D on gene expression. Effects of actinomycin D (**A**), epirubicin (**B**) doxorubicin (**C**), ebselen (**D**) and Erk inhibitor (**E**) on NR0B1-Luc (solid line) and UbC-Renilla (dotted line) reporter activity. Data plotted as mean +/− SEM of triplicates. **F**: Quantitative RT-PCR was used to determine the abundance of NR0B1, TP53 and RPS26 mRNA after overnight treatment of A673 cells with the indicated concentrations of actinomycin D. Results normalized to DMSO control. Data plotted as mean +/− SD of quadruplicates, and are representative of 3 independent experiments.

We co-transfected A673 Ewing sarcoma cells with NR0B1-Luc and UbC-RL reporters and treated cells with the compounds that scored positive in the AlphaScreen assay. For actinomycin D, the IC_50_ for inhibition of NR0B1 activity was 2.8 nM, whereas inhibition of UbC promoter activity was 14 nM (Figure 2A). In contrast, other compounds such as epirubicin (Figure 2B) and doxorubicin (Figure 2C) demonstrated no separation for inhibition of the NR0B1 and UbC reporters. Even though Erk inhibitor and ebselen demonstrated preferential inhibition of EWS-FLI binding to its target DNA *in vitro* (Figure 1, Table 1), both modulated the NR0B1 and UbC reporters equally in intact cells (Figure D and E). Thus, actinomycin D was the only compound that demonstrated preferential disruption of EWS-FLI1 binding *in vitro* and reporter activity in cells.

We next examined the effects of actinomycin D on the endogenous expression of NR0B1. As controls, we examined the expression of 2 transcripts that are not EWS-FLI1 targets (p53 and RPS26). Consistent with the reporter assay results (Figure 2A), expression of endogenous NR0B1 mRNA was preferentially attenuated at low concentrations of actinomycin D (5 -10 nM, Figure 2F). At higher concentrations, expression of NR0B1 and control transcripts were similarly attenuated (50 nM, Figure 2F). These results suggest that actinomycin D preferentially inhibits EWS-FLI1 in cells, however the window between this preferential effect and global inhibition of transcription is narrow (<10-fold).

### Global inhibition of EWS-FLI1 Activity

**Figure 3:**
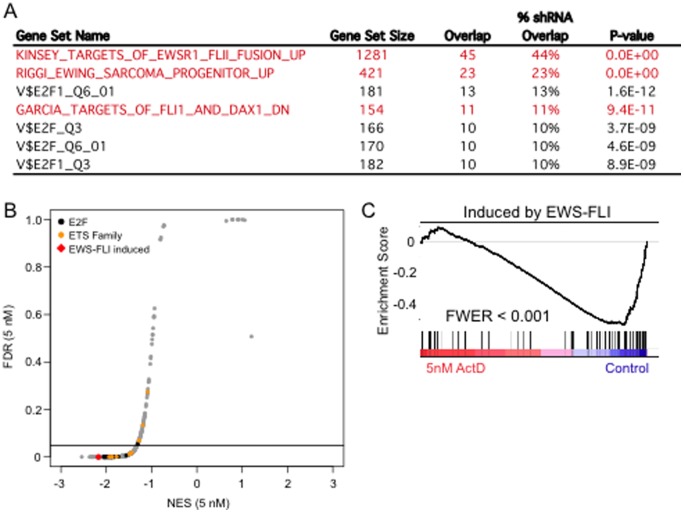
Effects of actinomycin D on EWS-FLI1 target gene expression. **A.** Genes down-regulated at least 2-fold by shRNA against EWS-FLI (adjusted p-value <0.01) were used to interrogate curated (C2 pathways, C5 gene ontology) and predicted (C3 transcription factor) gene sets. Shown are overlapping gene sets that encompassed at least 10% of the 102 EWS-FLI signature genes represented in MSigDB. **B.** Plot of normalized signature enrichment score vs FDR from GSEA using transcription factor signatures to distinguish cells treated with 5 nM actinomycin D from control. Subsets of signatures for E2F and ETS-family members are highlighted for comparison with gene sets induced or repressed by EWS-FLI. **C.** GSEA plot showing the down-regulation of EWS-FLI target genes by treatment with 5 nM actinomycin D.

To determine if the effects of actinomycin D on EWS-FLI1 target genes extended beyond NR0B1, we defined a set of EWS-FLI1 regulated genes by shRNA depletion of EWS-FLI1 in A673 cells. Cells were harvested and divided into two parts for Western-blot to determine protein levels (Figure S2) and RNA extraction for gene expression profiling 48 hrs after infection with a FLI1-targeting shRNA virus or a LacZ-targeting shRNA virus as a control. Genes whose expression was reduced at least 50% after knock-down of EWS-FLI1 (Table S1) were collated to form a gene set of EWS-FLI1 activated genes in A673 cells. A comparison of this gene signature to gene sets (C2 - curated and pathway gene sets, C3 - predicted transcription factor targets, and C5 - functional annotations) available from the Molecular Signatures Database (MSigDB) revealed strong enrichment for previously published EWS-FLI1 target gene sets (Figure 3A, limited to gene sets encompassing at least 10% of our EWS-FLI induced signature). Enrichment for E2F target genes was likely due to the anti-proliferative effects of EWS-FLI1 depletion.

We used Gene Set Enrichment Analysis (GSEA) to determine if treatment with low concentrations of actinomycin D resulted in a coordinated repression of EWS-FLI1-activated genes, effectively mimicking suppression by shRNA. The gene set comprised of EWS-FLI1-induced genes was among the curated signatures most robustly down-regulated by treatment with 5 nM actinomycin D (Figure 3B, red diamond). Consistent with the enrichment of E2F targets among EWS-FLI1-induced genes we noted in the shRNA study above (Figure 3A), we also found down-regulation of this class of signatures in A673 cells treated with low-dose actinomycin D (Figure 3B, black circles). Indeed, EWS-FLI1-induced genes which are highly expressed in control-treated A673 cells are repressed to a significant degree by 5 nM actinomycin D (Figure 3C). These results demonstrate that low concentrations of actinomycin D not only attenuates expression of NR0B1 (Figure 2D), but generally suppresses EWS-FLI1 mediated gene expression (Figure 3C).

### Disruption of EWS-FLI1 Binding to Chromatin

**Figure 4:**
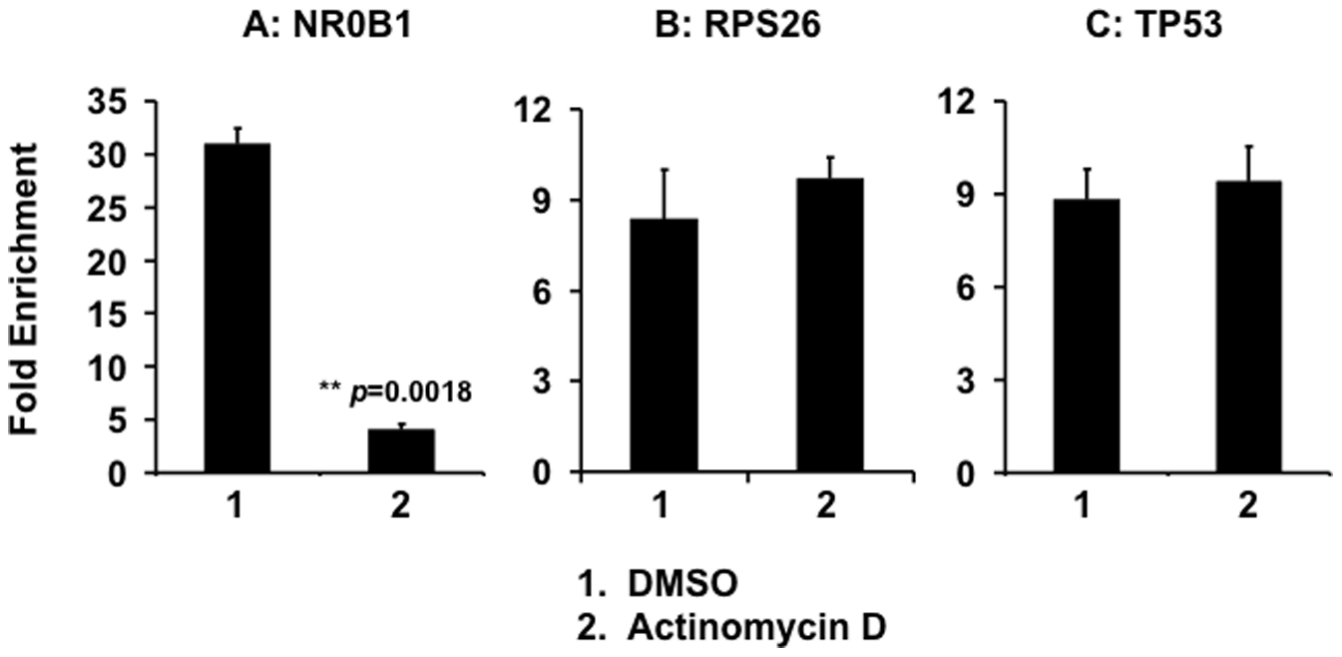
Effects of actinomycin D on the binding of EWS-FLI1 to the NR0B1 promoter. EWS-FLI1 was immunoprecipiatated using a FLI1 antibody, and quantitative PCR used to determine binding to NR0B1, RPS26, and p53. Data expressed as fold-enrichment over normal IgG control ChIP. Data plotted as mean +/− SD of duplicates are representative of 3 independent experiments.

We used chromatin immunoprecipitation coupled with quantitative PCR (ChIP-qPCR) to assess effects of actinomycin D on EWS-FLI1 binding to chromatin in cells. A673 cells were treated with either actinomycin D at 5 nM, or vehicle. After crosslinking, chromatin was sheared and EWS-FLI1-DNA complexes were immunoprecipitated using an anti-FLI1 antibody. We used primers flanking the EWS-FLI1-binding site in the promoter of the NR0B1 gene [15] to quantify binding of EWS-FLI1. We used RPS26 and TP53 as control loci since neither are bound by EWS-FLI [15] and we have shown that RPS26 and TP53 transcript levels were unaffected by actinomycin D treatment (Figure 2F). Without actinomycin D treatment, binding of EWS-FLI1 to the NR0B1 promoter was enriched 31-fold by comparison to IgG controls (Figure 4A, DMSO). Treatment of cells with actinomycin D reduced binding of EWS-FLI1 to the NR0B1 promoter to 4-fold (*p* = 0.0018; Figure 4A). Actinomycin D treatment did not affect either RPS26 or TP53 loci (Figure4 B–C). These data demonstrates that low concentrations of actinomycin D blocks the interaction of EWS-FLI1 with the NR0B1 promoter within intact cells.

### Comparative Sensitivity to Actinomycin D

**Figure 5:**
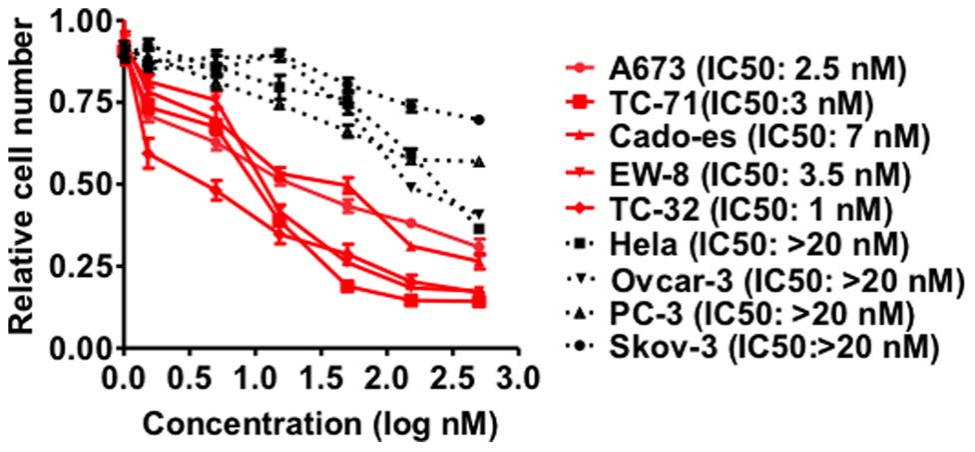
Growth effects of actinomycin D on Ewing sarcoma and other cancer cell lines. **Cells were treated with DMSO or actinomycin D for 44 hours.** Viable cell number was determined with a MTT assay. Data plotted as mean +/− SD of sextuplets and are representative of 3 independent experiments.

The studies above suggest that at low concentrations of actinomycin D, the binding of EWS-FLI1 is preferentially displaced from DNA. To determine if cells driven by EWS-FLI1 are more sensitive to actinomycin D, we assessed cell growth and viability using a MTT assay after 44 hrs exposure to actinomycin D. Although all cell lines were affected by actinomycin D, the Ewing sarcoma cell lines were as a group more sensitive by comparison to other cell lines tested (Figure 5). These results are consistent with the biochemical and cellular finding that low concentrations of actinomycin D preferentially disrupts EWS-FLI1 in Ewing sarcoma cells.

## Discussion

The EWS-FLI11 oncoprotein drives the development of Ewing sarcoma and is a propitious therapeutic target. In order to identify compounds that disrupt the binding of EWS-FLI1 to its cognate target genes, we develop a high throughput biochemical assay and used it to screen 5,200 small molecule bioactive compounds. Not surprisingly, we found that a number of DNA-binding agents disrupted the binding of EWS-FLI1 to DNA, and that most also disrupted the binding of p53 to DNA, indicating a lack of specificity. Actinomycin D was the only compound that demonstrated preferential blockade of EWS-FLI1 binding in biochemical and cell-based assays. Previous studies using a variety of methods including NMR [21], [25], [27], [28], [29], DNA footprinting [22], [23], [26], and X-ray diffraction [24], [30] have demonstrated sequence-specific binding of actinomycin D to dGpC, ATGCAT and T(G)nT where *n* varies from 1 to 4 guanine residues [31]. While none of these known sequence preferences correspond to a cognate EWS-FLI1 binding sequence, these results demonstrate that actinomycin D binds to different DNA sequences with different affinities, suggesting a possible explanation for the preferential disruption of EWS-FLI1 binding. Specifically, low concentrations of actinomycin D may occupy the highest affinity sites (e.g., ATGCAT), the cognate EWS-FLI1 binding sequences may represent moderate affinity sites, whereas high concentrations of actinomycin D would occupy low affinity sites resulting in widespread blockade of transcription factor binding to DNA.

The preferential disruption of EWS-FLI1 binding at low concentrations of actinomycin D is apparent not only in biochemical assays, but also in cell-based assays. Using gene expression profiling, we can demonstrate that the disruption of EWS-FLI1 mediated gene expression is not limited to the NR0B1 gene, but is widespread across all EWS-FLI1 target genes. We suspect that this widespread disruption of EWS-FLI1 activity may be responsible for the heightened sensitivity of Ewing sarcoma cells to actinomycin D. These results are reminiscent of prior studies demonstrating that mithramycin and ET-743 (trabectedin), both DNA binding agents, reduced expression of EWS-FLI1 downstream targets and inhibited the growth of Ewing sarcoma cells [11], [12].

Unfortunately, while actinomycin D concentrations can be precisely titrated *in vitro*, the use of actinomycin D *in vivo* is subject to pharmacokinetics whereby constant drug levels at a precise concentration are not possible to achieve. Therefore, these *in vitro* and cell-based studies are not readily translatable to *in vivo* utility. However, together these results demonstrate that DNA-binding agents do demonstrate differential specificity, likely due to preferential sequence binding affinities. These results suggest that compounds with even greater sequence specificity may be identified either through expanded screening (and counter-screening), or through medicinal chemical modification of existing DNA-binding agents (SAR for specific disruption of EWS-FLI1). Compounds would ideally have a wide therapeutic window between disruption of EWS-FLI1 binding and other transcription factors (specificity) and pharmacokinetic properties that allow clinically achievable drug levels within this therapeutic window. Identification of compounds with such properties may allow *in vivo* and clinical translation to specifically target EWS-FLI1 dependent tumors.

## Supporting Information

Figure S1 **NR0B1 reporter is driven by EWS-FLI1. A.** HeLa cells were transfected with both NROB1-firefly luciferase and UbC-renilla reporters, and with or without EWS-FLI1 expression vector. Data plotted as mean +/− SD of triplicates. **B.** HeLa cells were transfected with NROB1-firefly luciferase reporter and with a graded amount of EWS-FLI1 expression vector. Data plotted as mean +/− SD of triplicates. **C.** Ewing sarcoma A673 cells were transfected with NR0B1-Luc reporter with either EWS-FLI1 binding sites deleted (1) or intact (2). Data plotted as mean +/− SD of triplicates.
(TIF)

Figure S2 **shRNA knock-down of EWS-FLI1.** A673 cells were infected with lentiviruses encoding FLI1-targeting shRNA or a control LacZ-targeting shRNA for 48 hours. Western blot was used to assess abundance of EWS-FLI1 compared to beta-actin as a loading control.
(TIF)

Table S1 (DOC)
