# Supplementary figures and images for: Differential Disruption of EWS-FLI1 Binding by DNA-Binding Agents
Source: PLoS One. 2013 Jul 22;8(7):e69714. doi: 10.1371/journal.pone.0069714 (PMC3718762; doi:10.1371/journal.pone.0069714)

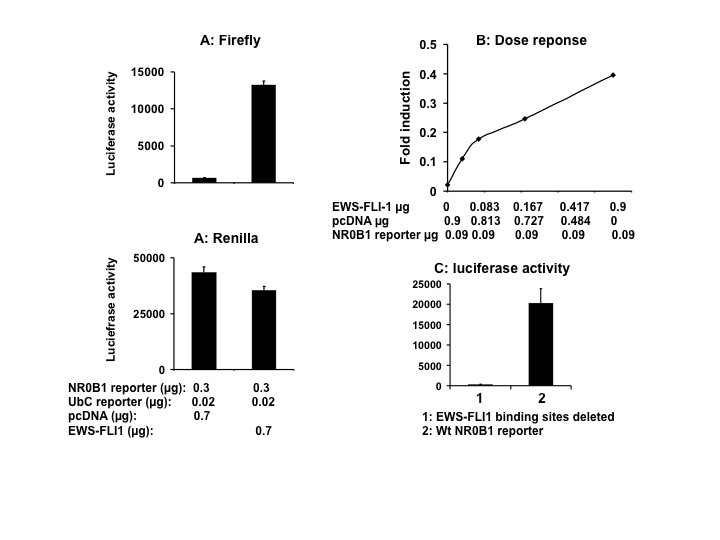

Supplement: Figure S1 — NR0B1 reporter is driven by EWS-FLI1. A. HeLa cells were transfected with both NROB1-firefly luciferase and UbC-renilla reporters, and with or without EWS-FLI1 expression vector. Data plotted as mean +/− SD of triplicates. B. HeLa cells were transfected with NROB1-firefly luciferase reporter and with a graded amount of EWS-FLI1 expression vector. Data plotted as mean +/− SD of triplicates. C. Ewing sarcoma A673 cells were transfected with NR0B1-Luc reporter with either EWS-FLI1 binding sites deleted (1) or intact (2). Data plotted as mean +/− SD of triplicates. (TIF) [file pone.0069714.s001.tif]

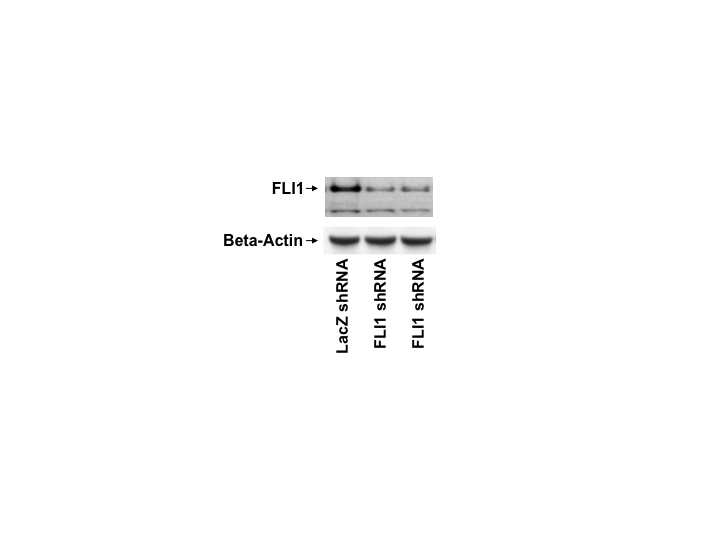

Supplement: Figure S2 — shRNA knock-down of EWS-FLI1. A673 cells were infected with lentiviruses encoding FLI1-targeting shRNA or a control LacZ-targeting shRNA for 48 hours. Western blot was used to assess abundance of EWS-FLI1 compared to beta-actin as a loading control. (TIF) [file pone.0069714.s002.tif]
