# Supplementary material for: Differential Disruption of EWS-FLI1 Binding by DNA-Binding Agents
Source: PLoS One. 2013 Jul 22;8(7):e69714. doi: 10.1371/journal.pone.0069714 (PMC3718762; doi:10.1371/journal.pone.0069714)
Supplement: Table S1 — (DOC) [file pone.0069714.s003.doc]

**Table S1. Genes downregulated >50% with shRNA depletion of EWS-FLI1**

| ABCC4 | GPR160 | RIBC2 |
| --- | --- | --- |
| ACTN2 | GRM2 | RIMBP3B |
| ADRB3 | HAPLN1 | RNF148 |
| ALK | HELLS | ROR1 |
| ARHGAP20 | IGF1 | SCNN1G |
| ATP1A3 | IRX3 | SERPINB13 |
| BARHL2 | JPH1 | SLC1A4 |
| C13orf18 | KCNA2 | SLC26A2 |
| C1orf161 | KCNE3 | SLC35F1 |
| CCNE1 | KCNN1 | SLC43A1 |
| CDCA7 | KIT | SMA4 |
| CDH12 | KMO | SNORA75 |
| CER1 | LBH | SNORD20 |
| CLSPN | LOXHD1 | SNORD22 |
| CLUL1 | LRFN5 | SNORD26 |
| CYP4F22 | MAP7D2 | SNORD27 |
| DCDC2 | MBD3L2 | SNORD28 |
| DHRS3 | MCM10 | SNORD44 |
| DNA2 | MCM2 | SNORD75 |
| DPF3 | MORC1 | SNORD78 |
| DPYSL5 | MTNR1A | SNORD79 |
| DTL | MYB | ST8SIA5 |
| E2F1 | NCBP1 | STEAP1 |
| E2F2 | NGFR | SV2B |
| EFNB2 | NKX2-2 | SYNPR |
| EXO1 | NPY1R | TCF19 |
| FAM111B | NPY5R | THSD3 |
| FAM123A | NR0B1 | TMEM84 |
| FAM136A | OR4E2 | UHRF1 |
| FEZF1 | ORC1L | UNC5A |
| FGF18 | PCNA | UNG |
| FLI1 | PEG3 | WDR76 |
| FLJ14213 | PHOSPHO1 | WEE1 |
| FLJ38379 | POLD3 | XRCC2 |
| GFRA2 | RASGEF1B | ZMAT4 |
| GPR128 | RGS7BP |  |
